# Bandgap Tunable Two-Step Vapor-Deposited Perovskite Absorbers for Perovskite-Silicon Tandem Solar Cells

**DOI:** 10.1021/acsenergylett.6c00156

**Published:** 2026-03-13

**Authors:** Austin G. Kuba, Kerem Artuk, Mostafa Othman, Deniz Turkay, Chiara Ongaro, Michele Debastiani, Quentin Guesnay, Mohammad Reza Golobostanfard, Maryamsadat Heydarian, Oliver Fischer, Martin C. Schubert, Florent Sahli, Yohann Ansel, Quentin Jeangros, Aïcha Hessler-Wyser, Christophe Ballif, Christian M. Wolff

**Affiliations:** † Ecole Polytechnique Fédérale de Lausanne (EPFL), Institute of Electrical and Microengineering (IEM), Photovoltaics and Thin-Film Electronics Laboratory, 2000 Neuchâtel, Switzerland; ‡ Centre Suisse d’Electronique et de Microtechnique (CSEM), 2002 Neuchâtel, Switzerland; § Smart Energy Materials, Department of Chemistry, University of Turku, Henrikinkatu 2, 20500, Turku, Finland; ∥ Fraunhofer Institute for Solar Energy Systems, Heidenhofstrasse 2, 79110 Freiburg, Germany; ⊥ Chair for Photovoltaic energy Conversion, Department of Sustainable Systems Engineering INATECH, University of Freiburg, Emmy-Noether-Str. 2, 79110 Freiburg, Germany

## Abstract

We report a two-step thermal evaporation–close space sublimation deposition method for tunable bandgap lead halide perovskites with single junction solar cell efficiencies exceeding 20%. These absorbers are integrated into perovskite–silicon tandem solar cells with stabilized efficiencies of >29%.

## Graphical abstract

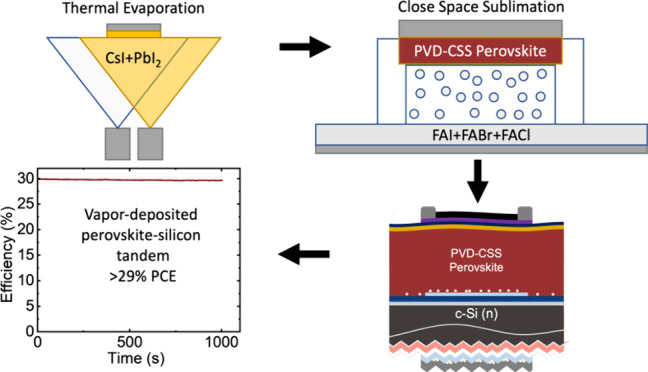

Photovoltaic energy generation continues to drive down the cost of electricity. As single junction (SJ) silicon solar cells approach their practical limits,^1^ next generation concepts are needed for continued progress. The most successful route to overcoming these limits is the use of multijunction solar cells.^2,3^ Perovskite–silicon tandem solar cells (TSCs) have recently seen a dramatic rise in power conversion efficiency (PCE), achieving up to 34.9%.^2^ Lead halide perovskites (LHPs) have continued their progress in stability to the point that encapsulated devices can pass damp heat and have improved in accelerated aging tests^4^ meaning they may soon find broader adoption in the market. However, most available reports focus on solution processes, which are still under debate regarding their suitability for commercialization on an industrial scale. Despite being rare in the literature, vapor-processed LHPs have shown promise regarding scalability and stability.^5,6^ They have also attained ∼26% PCE^7^ in SJ solar cells, approaching their solution-processed counterparts. However, there are still few reports of TSCs produced with fully vapor-processed LHP absorbers.^8–11^ A recent breakthrough in coevaporated LHP processing has achieved >30% stabilized PCE^12^ but two-step vapor-processed TSCs are still limited to <27% ^11,13^ to date. One technique receiving increasing attention is two-step physical-vapor-deposition close-space-sublimation (PVD-CSS, Supporting Note 1). We recently reported a PVD-CSS process capable of creating p-i-n solar cells with a broad range of bandgaps and SJ devices with efficiencies approaching 17%.^14^ In this process, an inorganic lead halide-cesium halide template is deposited using thermal evaporation and then converted to a LHP by reaction with mixed organohalide vapors in a CSS configuration (Supporting Note 2). Here, we adapt this process, show the absorbers are compatible with standard passivation treatments, and incorporate these absorbers into LHP-silicon TSCs achieving >29% PCE from stabilized maximum power point (MPP) tracking.

First, a series of additives were screened. FACl produced the highest photoluminescence (PL) quantum yield (Figure S1) and has already shown promise in CSS reactions using pure formamidimium iodide (FAI)^11^ so it was chosen for the rest of the work. Next, the bandgap tunability was investigated by varying the ratio of FAI to formamidinium bromide (FABr) with a constant formamidinium chloride (FACl) addition using coevaporated CsI:PbI_2_ or CsBr:PbI_2_ templates. The achievable range of bandgaps is shown in Figure 1a. The bandgap shows excellent tunability from 1.55 to 1.90 eV, even using pure iodide templates (spectrophotometry and PL in Figure S2). The conversion time was set to 20 min at 175 °C, and the organohalide mass addition was tuned to optimize conversion and device performance (Figures S3 and S4). SJ solar cells with an area of 0.1 cm^2^ were made with CsI:PbI_2_ templates and FAI/(FAI+FABr) values of 1.0, 0.8, and 0.6 (by mass, see Supporting Note 3). The current density–voltage (JV) performance is shown in Figure 1b (JV parameters are given in Table S1). External quantum efficiency (EQE) is shown in Figure 1c. X-ray diffraction (XRD) is shown in Figure S5 and scanning electron micrographs (SEMs) are shown in Figure S6.

**Figure 1:**
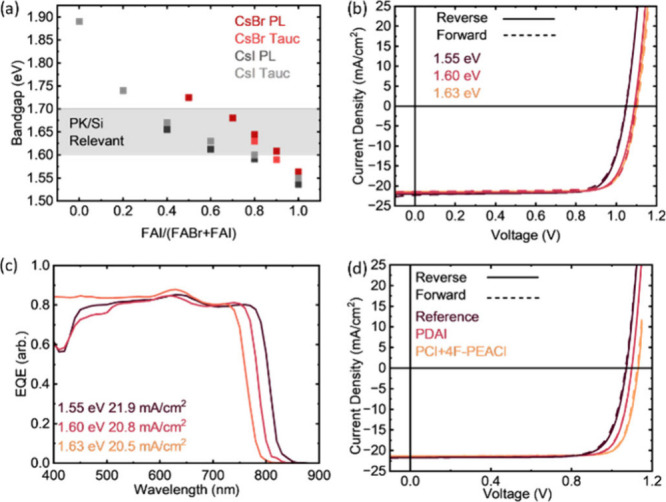
(a) Bandgap tunability for varying Cs source (CsI vs CsBr) with varying values of FAI/(FAI+FABr). (b) JV and (c) EQE for devices with varying bandgaps achieved by using CsI in the template and varying FAI/(FAI+FABr) from 1.0 to 0.8 to 0.6 by mass. (d) Study of surface passivation for the 1.60 eV absorber.

The 1.60 eV absorber with a short circuit current density of ∼ 21 mA/cm^2^ was selected for further testing and integration into TSCs. This was expected to create good current matching with a slight bottom cell limitation based on our previous work showing a cumulative current density of 40–41 mA/cm^2^ in LHP-silicon TSCs with flat front, rear textured silicon bottom cells.^15^ SJ devices incorporating no passivation, propane-1,3-diammonium iodide (PDAI_2_),^16^ or bimolecular passivation of 4F-phenylethylammonium chloride (4F-PEACl) and piperazinium chloride (PCl)^17^ are shown in Figure 1d, with bimolecular passivation achieving a champion efficiency of >20% without optical management. Across two batches of six films with different passivation conditions, excellent repeatability and yield were shown (Figure S7).

Six LHP-silicon TSCs with an active area of 1 cm^2^ were made with PDAI_2_ passivation. Good consistency across the batch was shown, with a mean PCE of 29.1% ± 0.6 (*n* = 6, average of forward and reverse scans) and a minimum of 27.8%. Cross-section SEM is shown in Figure 2a and (top-view Figure S8), and a schematic of the devices is shown in Figure 2b. The champion device reached 30.7% PCE from the reverse JV scan (Figure 2c) with a MPP efficiency of 29.8% for the first 300 s and minor degradation to 29.7% over 1000 s of tracking unencapsulated in air (Figure 2d). The champion device also showed good shelf stability, retaining 99% of its MPP efficiency over 134 days of storage in a N_2_ environment (Figure S9). Subcell selective EQE of the champion device is shown in Figure 2e. Current mismatch measurements confirm that the top cell produces 1 mA/cm^2^ more than the bottom cell (Figure S10) which can be improved for marginal current gains by increasing the FABr:FAI ratio to fine-tune the LHP bandgap. Four of the TSCs were cross-tested at Fraunhofer ISE after 3 weeks of shelf storage for an independent confirmation of the device performance. The JV parameters of the champion device and the selected devices are given in Table S2 and Figure S11 at both institutes. The relative error in the PCE for the champion device is 2% from MPP tracking (Figure S12). Subcell selective PL-based i*V*_OC_ images^18^ (Figure 2f,g) of the champion TSC show good uniformity with small point-like defects in the top cell and an average i*V*_oc_ of 1.158 V for the top cell and 0.724 V from the bottom cell, in good agreement with the measured *V*_oc_ from JV. To explore the suitability of this process for TSCs on textured silicon bottom cells, we made one batch of LHP-silicon TSCs on textured Si with pyramid sizes ∼1 μm (SEM shown in Figure S13). The champion device achieved a maximum PCE of 28.3% from reverse JV (Figure S14) with *V*_oc_ = 1.86 V and *J*_sc_ = 20.4 mA/cm^2^ but low FF = 75%. Sister devices on ohmic textured Si substrates show that the low FF is likely related to low shunt resistance (Figure S14), which must be improved in future work to enable high efficiency textured TSCs.

**Figure 2:**
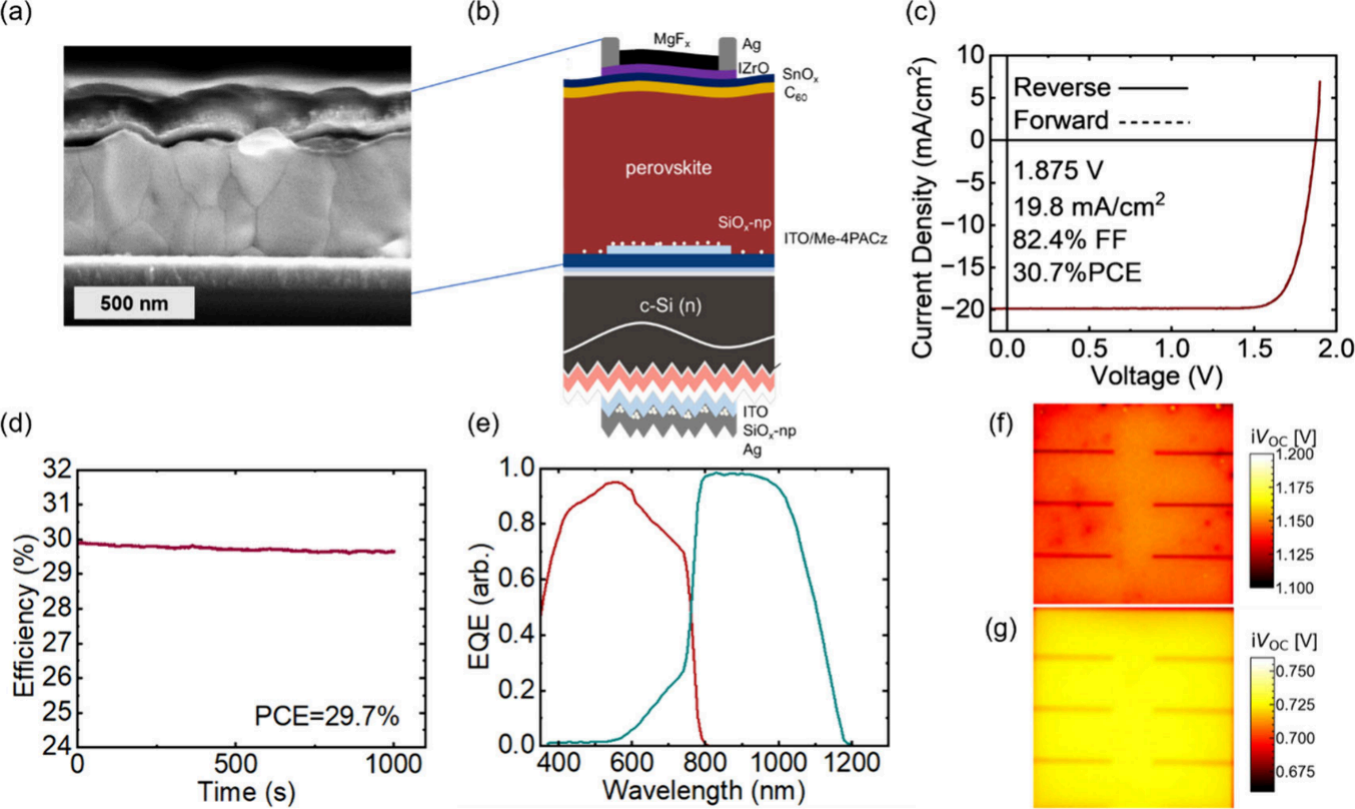
(a) Cross section SEM of a LHP-silicon tandem device produced in this work focused on the LHP top cell. (b) Schematic of a tandem device. (c) JV curve of the champion tandem solar cell produced in this work. (d) MPP tracking of the champion device for 1000 s unencapsulated in air. (e) Subcell selective EQE for the top and bottom cell. (f) Subcell selective PL-based i*V*_OC_ images of the LHP top cell and (g) silicon bottom cell (full image width 1 cm in both cases).

In conclusion, we report the fabrication of two-step vapor-deposited LHP-silicon tandem solar cells with good scalability potential and efficiencies >29% through a PVD-CSS process. This report represents a significant advance in the performance of two-step vapor-deposited LHP-silicon tandem solar cells. These results motivate further studies into the potential advantages or disadvantages that vapor-deposited LHPs may have, in terms of stability and scalability, for both single junction and multijunction solar cells, and whether their efficiencies can further approach solution-processed solar cells in record efficiency.

## Supplementary Material



## Notes

Experimental details and additional data on LHP films, single junction, and tandem solar cells (PDF)
