# Supplementary material for: Bandgap Tunable Two-Step Vapor-Deposited Perovskite Absorbers for Perovskite-Silicon Tandem Solar Cells
Source: ACS Energy Lett. 2026 Mar 13;11(4):3164–7. doi: 10.1021/acsenergylett.6c00156 (PMC13078313; doi:10.1021/acsenergylett.6c00156)
Supplement: Supplementary file 1 [file nz6c00156_si_001.pdf]

Supporting Information for:

Bandgap Tunable Two-Step Vapor-Deposited Perovskite Absorbers for Perovskite-Silicon Tandem Solar Cells

Austin G. Kuba[1*], Kerem Artuk[1], Mostafa Othman[1], Deniz Turkay[1], Chiara Ongaro[1], Michele Debastiani[2], Quentin Guesnay[1], Mohammad Reza Globostanfard[1,3], Maryamsadat Heydarian[4], Oliver Fischer[4,5], Martin C. Schubert[4], Florent Sahli[2], Yohann Ansel[2], Quentin Jeangros[2], Aïcha Hessler-Wyser[1], Christophe Ballif[1], Christian M. Wolff[1*]

[1] Ecole Polytechnique Fédérale de Lausanne (EPFL), Institute of Electrical and Microengineering (IEM), Photovoltaics and Thin-Film Electronics Laboratory, 2000 Neuchâtel, Switzerland

[2] Centre Suisse d'Electronique et de Microtechnique (CSEM), 2002 Neuchâtel, Switzerland

[3] Smart Energy Materials, Dept of Chemistry University of Turku, Henrikinkatu 2, 20500, Turku, Finland

[4] Fraunhofer Institute for Solar Energy Systems, Heidenhofstrasse 2, 79110 Freiburg, Germany

[5] Chair for Photovoltaic energy Conversion, Department of Sustainable Systems Engineering INATECH, University of Freiburg, Emmy-Noether-Str. 2, 79110 Freiburg, Germany

*agkuba@gmail.com, christian.wolff@epfl.ch

Supporting note 1. A brief review on close space sublimation of perovskites. Close space sublimation (also called close space vapor transport and sometimes by the more general term vapor solid reaction) is a process where the source material is separated from the substrate by a small space as compared to thermal evaporation (<1cm vs >10cm). Because the spacing is lower, higher pressures can be used while achieving sufficient deposition rates, which can simplify equipment design and decrease capital expenditures. For hybrid perovskites, organohalides and lead halides have widely diverging volatilities, leading to the common use of two-step processes. In most reports, the deposition of organohalides is first performed by spin coating, thermal evaporation, or CSS, then this inorganic template is converted to a perovskite using an organohalide vapor (CSS conversion, wherein a vapor solid reaction occurs). CSS of the inorganic template is still rare in the literature.

The first reports of CSS used solution deposition to create the inorganic template, and then used a solution of organohalides on glass to form the vapor source (solution-CSS). Li, Ho, Wong, and Kwok used such a process to fabricate 16% efficient n-i-p solar cells in 2015[14]. Guo et al demonstrated 16% efficient p-i-n solar cells in 2016[15]. By alloying the solution processed template with PbI_2:$PbBr_2$:$PbCl_2$, >20% n-i-p solar cells were demonstrated in 2020[16].

Zu et al. demonstrated an early report of a PVD-CSS process using coevaporation of CsCl and PbCl2 and conversion directly in contact with solid MAI powder to demonstrate >20% efficiency, demonstrating that cesium content was critical to device performance.[17] Luo et al demonstrated coevaporated CsBr:PbI2 with an FAI:FACl conversion step in 2018 achieving >17%.[18]

Two-step CSS where both CSS deposition of PbI_2 from a melted ingot and then CSS conversion with solid MAI powder was reported by Harding et al in 2020[19]. Kuba et al used this two-step close space sublimation process to examine the growth dependence on different substrate layers and the effect of residual lead iodide on device performance[20], all vapor processed cells using thermally evaporated CuPC[21], and to examine the stability of two-step vapor deposited solar cells using fullerene ETLs[22].

Beginning in 2022, the technique has received increasing interest, with the bulk of demonstrations focusing on PVD-CSS. Several impressive results have been achieved by the groups of Zhiliang Ku and Yi-Bing Chen among others. They demonstrated PCEs of >20% in n-i-p cells with tunable bandgap between 1.54 and 1.60 with conversion times as fast as three minutes by lowering the substrate-source distance to 0.2 mm[23]. Pretreatment of the inorganic template[24,25] and post-treatment of the perovskite film[26–30] buried interface engineering[31–33] and additive/process engineering[3,34–36] have recently been

reported to improve efficiencies to the 20-22% level for both n-i-p and p-i-n perovskite solar cells. Guesnay et al. demonstrated precise bandgap tunability using a PVD-CSS process achieving nearly 17% PCE[1] showing the potential of the process for tandem solar cells with optimized bandgaps. Rodkey et al used a PVD-CSS process with a reusable solid FAI compressed pellet source to with n-i-p efficiencies of >18%[37]. Gomar-Fernandez et al. used a similar process with a MAI powder source to demonstrate parallel processing of multiple substrates achieving PCE of up to 18.8% in p-i-n with excellent stability[38]. The first ever demonstration of a CSS process to produce perovskite/silicon tandem solar cells was reported in 2025[39] achieving >18% in single junction and 26.3% from MPPT. Notably, examinations of the utility of surface passivation in the field are still largely absent as well as high efficiency two terminal perovskite/silicon tandem solar cells. In this work we show that this versatile process is compatible with current standard surface passivation treatments and can be used to make efficient tandem solar cells with >29% efficiency and the potential for future improvement.

Supporting note 2. On choice of CSS reaction scheme. For eventual success of this technique on an industrial scale, we note that careful thought must be put into the organohalide conversion source. Currently, there are two main approaches. In one method, organohalides are dissolved in solution then spread on a substrate such as glass or silicon and dried before use as the organohalide source. The second iteration is the use of dry organohalide powders or pellets as sources.

To our knowledge the first method was first demonstrated in 2016[14,15] and the second was first demonstrated in 2017[17]. The authors of this work have used both approaches[1,20]. In previous work by the authors of this manuscript, dry MAI powder sources were used that were reusable for hundreds of runs[19,20,22,40]. This reusability has also been demonstrated in FAI pellet sources[37] and the authors of this work have also used FAI powder sources for >1year periods with stable conversion behavior (data not shown). The reusability of these types of sources is a clear advantage, industrially, and they are fully suitable at least for medium bandgap perovskites with E_G<1.6eV.

However, a difficulty arises when fabricating mixed halide (MA-free) FA based perovskites. The volatility of FABr and FAI are notably different[41]. In the same reactor used for this work, when using pure FAI powder enclosed in a Teflon cube, conversion was accomplished within ~20 minutes at 175C but when using pure FABr powder, full conversion was accomplished in <20 minutes at 110C, and the FABr powder source liquified and seemingly boiled, leading to damage of the template (shown below).

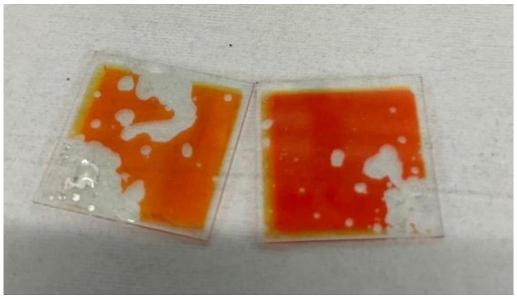

Considering these factors, unless the solid-state alloy of FAI and FABr shows notably different properties than the single powders, we anticipate notable difficulties using FABr in any bulk dry powder source, as it is likely FABr will evaporate more quickly than FAI and the source composition may drift over time. It may be possible to use mixtures of FAI, MAI, and MABr to create reusable bandgap tunable sources, as their volatilities are more similar[41], but it still remains possible that over time the I:Br ratio may drift due to any existing difference in volatility. Thus, in this work we use single use sources where the organohalide solution is deposited on a silicon wafer. Single use sources also have the advantage that the reaction can be precisely tuned by changing the organohalide mass addition to the source, to result in

under-reacted, well-converted, or over-reacted films[1] rather than relying on optimization of temperature and time in dry powder sources[20]. We note that glass is also commonly used as a vapor deposition source e.g. in the work from Ku group, but silicon has higher thermal conductivity and lower thermal mass which may lead to a faster and more even conversion depending on the heating geometry of the reactor. Another possible method enabling tandems from dry powders would be to use vapor exchange where Br rich templates are partially converted with FABr then FAI to tune the bandgap[3], but the exact controllability of the bandgap using this method is not yet reported. We note that we dispense 0.4 ml of a nominally 10 mg/ml organohalide solution (12 mg/ml including FACl), meaning just 4 mg of FAI+FABr and 0.8 mg of FACl are consumed per substrate. This shows the high level of material efficiency inherent in this process.

Supporting note 3. Regarding terminology for the organohalide conversion solution. In this work, we use single-use organohalide vapor sources consisting of 0.4 ml of organohalide solution deposited onto a freshly cleaned rough-cut silicon wafer. The reaction temperature is fixed at 175°C and the time is fixed at 20 minutes. To tune the extent of reaction, the mass addition of organohalides to the source is changed by changing the concentration of the solution deposited on the wafer source. In this work, we report the ratio of FAI to FABr in the form of FAI/(FAI+FABr). FACl is regarded as an additive and significant Cl is not expected to remain in the perovskite due to its high volatility and limited miscibility in the perovskite lattice (an assumption that should be further tested in future work). Thus, when discussing the concentration or mass addition of organohalides, we refer to the mass of FAI+FABr neglecting FACl additive. A table is presented below which shows the equivalent molar ratios to the mass ratios for easy reference.

For illustrative purposes, the optimal solution for converting a 300 nm PbI_2 30 nm CsI inorganic template in this work on flat tandems contained 8 mg/ml FAI, 2 mg/ml FABr, 2 mg/ml FACl. Under the framework above, this leads to a total FAI+FABr mass addition of 4 mg with 0.8 FAI/(FAI+FABr) ratio over the surface of the 4-inch wafer. We note that we do not consider the effect of volume expansion and contraction in the calculation of the mass ratio which may lead to minor alterations of the true ratio. More details on the preparation of the organohalide source are detailed in the next section.

Mass FAI (mg/ml)	Mass FABr (mg/ml)	Total mass (mg/ml)	Mass ratio FAI/(FAI+FABr)	Molar ratio FAI/(FAI+FABr)
50	0	50	1.00	1
45	5	50	0.90	0.87
40	10	50	0.80	0.74
35	15	50	0.70	0.63
30	20	50	0.60	0.52
25	25	50	0.50	0.42
20	30	50	0.40	0.33
15	35	50	0.30	0.24
10	40	50	0.20	0.15
5	45	50	0.10	0.07
0	50	50	0.00	0.00

Experimental details

Close space sublimation conversion of cesium halide/ lead halide template to perovskite:

For the conversion of the evaporated lead halide template to a perovskite, we followed a similar process to our previous report[1]. The vacuum chamber has a heated substrate table controlled to 175°C. The base pressure of the vacuum chamber is 0.3 mBar. The walls of the chamber are heated by a glass fiber heating blanket set to 200°C to avoid condensation of vapors. The chamber is allowed to preheat under vacuum for at least two hours before deposition to reach a steady state.

To form the organohalide conversion solution, first 30 mg of FACl was dissolved in 3 ml ethanol. 1 ml of this solution was used to dissolve 50 mg FAI. In a separate vial, another 1 ml of the FACl solution was used to dissolve 50 mg FABr. These stock solutions were then mixed by volume ratio to obtain a mixed halide stock solution with the desired FAI/(FAI+FABr) ratio. This mixed halide stock solution was then diluted with ethanol to obtain the desired FAI+FABr concentration. The volume of solution with a certain FAI+FABr concentration determines the total FAI+FABr mass addition to the wafer surface, which in this work was fixed at 0.4 ml over the four-inch wafer surface.

We present as an example the optimal flat tandem recipe, which was also applied to the textured tandems. First, stock FAI/FACl and FABr/FACl solutions were prepared as above. 0.8 ml of concentrated FAI/FACl solution was mixed with 0.2 ml of concentrated FABr/FACl solution to obtain a stock solution with a FAI/(FAI+FABr) ratio of 0.8 and a concentration of "50 mg/ml" (neglecting volume expansion/contraction effects). Then 0.1 ml of the mixed organohalide stock solution was diluted with 0.4 ml ethanol to achieve a "10 mg/ml" conversion solution. 0.4 ml of this solution deposited onto the four-inch silicon wafer source leads to a FAI+FABr mass addition of 4 mg. We reiterate for emphasis and transparency that we refer to the concentrations as mg/ml, ignoring volume expansion/contraction effects, which may slightly affect the true molar ratio and concentration in the solution. Based on the species in the template and organohalide solution, the expected composition in the optimal tandems is $Cs_{0.13}FA_{0.87}Pb(I_{0.925}Br_{0.075})_3$ but the exact composition may be slightly different in the final film due to halide exchange processes[2,3]. A table showing the expected composition of different films over different template and FAI/FABr ratios assuming complete conversion is below neglecting halide exchange.

Alkali halide	Lead Halide	Volume ratio	FAI/(FAI+FABr) mass	FAI/(FAI+FABr) mol	Cs/Pb	x=I/(I+Br)
CsI	PbI2	0.1:1	1.0	1.000	0.13	1.000
CsI	PbI2	0.1:1	0.8	0.744	0.13	0.926
CsI	PbI2	0.1:1	0.6	0.522	0.13	0.861
CsI	PbI2	0.1:1	0.4	0.326	0.13	0.805
CsI	PbI2	0.1:1	0.2	0.154	0.13	0.755
CsI	PbI2	0.1:1	0.0	0.000	0.13	0.710
CsBr	PbI2	0.1:1	1.0	1.000	0.16	1.000
CsBr	PbI2	0.1:1	0.8	0.744	0.16	0.876
CsBr	PbI2	0.1:1	0.6	0.522	0.16	0.813
CsBr	PbI2	0.1:1	0.4	0.326	0.16	0.758
CsBr	PbI2	0.1:1	0.2	0.154	0.16	0.710
CsBr	PbI2	0.1:1	0.0	0.000	0.16	0.667

In this work, single-side polished four-inch silicon wafers were used for the organohalide source. The saw damaged side was used as the source. Before every deposition, the wafers were successively cleaned in IPA and DI water, followed by 15 minutes of UV-ozone cleaning to ensure a clean surface for deposition and enhance the wetting of the organohalide on the wafer surface. To form the organohalide source, 400 µl of the conversion solution was dispensed onto the unpolished side of the wafer and allowed to dry in room air for up to 5 minutes until all the ethanol evaporated. A visible organohalide salt film was left behind on the surface of the wafer. Separately, a 1 cm thick piece of Teflon machined to hold a 2.5 x 2.5 template with a 2.4 x 2.4 cm square hole in the center to allow vapor transport from the source to the film was preheated for 5 minutes under vacuum in the vacuum chamber. This sets a 1 cm spacing between the source and substrate. The preheated Teflon holder was removed, and the inorganic template was placed on top, template facing downwards. Next, the organohalide source was placed onto the heated table within the vacuum chamber, the Teflon holder with template was placed on top and the chamber was pumped down to initiate the reaction. The reaction was allowed to proceed for 20 minutes before the chamber was vented, and the converted perovskite was removed. No additional heating or washing steps were performed before finishing the devices (excluding passivation treatments where specified).

A picture of the CSS system is shown below. The system is the same as described in previous work on VTD[4] and CSS[1] of lead halide perovskites.

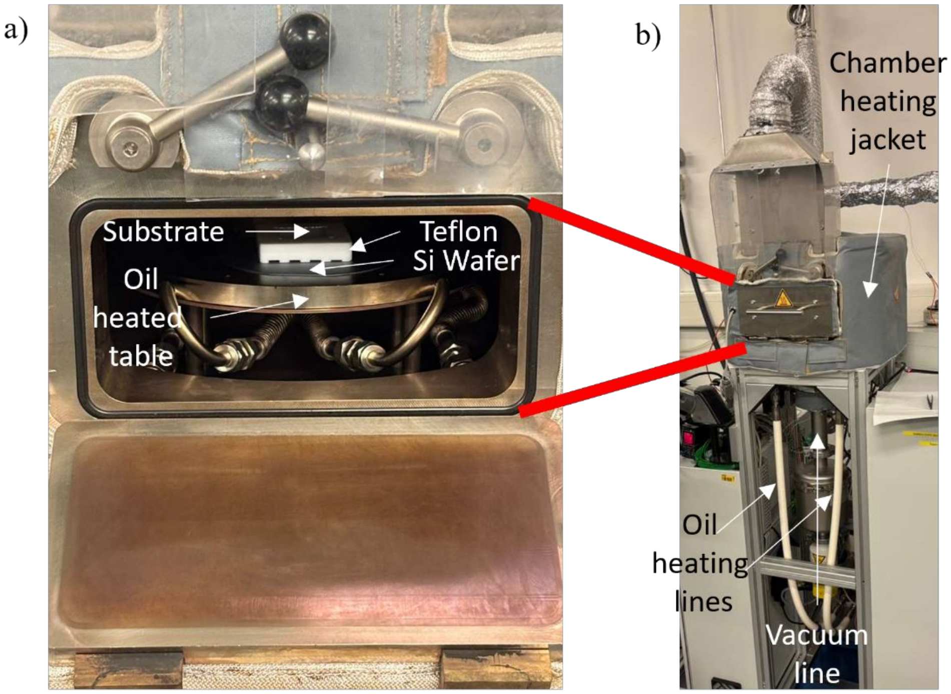

Solar cell fabrication

Single junction fabrication: ITO glass substrates were cleaned by successively sonicating in acetone, IPA, helmanex, and DI water solution for 10 minutes each. The cleaned substrates were uv-ozone treated for 15 minutes. Next, 150 µL of 1 mg/ml Me4PACz/ethanol was statically dispensed on the ITO and spun at 3000 rpm for 40s with a 300 rpm/s ramp rate before annealing for 10 minutes at 100°C. A 1 weight percent solution of SiO_x nanoparticles in ethanol solution was further diluted in ethanol to 0.1%. 150 µL of this solution was statically dispensed onto the ITO and spun at 3000 RPM for 40s with a 300 rpm/s ramp rate without further drying to improve shunt resistance[5]. Next, 30 nm of CsI is coevaporated with 300 nm of PbI_2 onto the substrate using a Lesker Mini Spectros evaporator to form an inorganic template.

This inorganic template film is converted with an organohalide vapor in a close space sublimation configuration[1] as described above. To complete the cell, 25 nm of C_{60} is thermally evaporated in a custom evaporator at a rate of 0.3 Å/s. Next, 225 cycles of atomic layer deposited SnO_x (95 degrees, 0.12 nm/cycle) is deposited using a PICOSUN R-200 Standard ALD system. Finally, 130 nm Ag is evaporated through a shadow mask at a rate of 1 Å/s.

Perovskite silicon tandem cell fabrication: For tandem solar cells, the ITO glass substrate is substituted with a silicon bottom cell and the solvent cleaning steps are omitted. The silicon bottom cell was UV-ozone treated for 15 minutes. Next, 150 µL of 1 mg/ml Me4PACz/ethanol was statically dispensed on the ITO and spun at 3000 rpm for 40s with a 300 rpm/s ramp rate before annealing for 10 minutes at 100°C. A 1 weight percent solution of SiO_x nanoparticles in ethanol solution was further diluted in ethanol to 0.1%. 150 µL of this solution was statically dispensed onto the ITO and spun at 3000 RPM for 40s with a 300 rpm/s ramp rate without further drying to improve shunt resistance[5]. Next, for flat tandems 30 nm of CsI is coevaporated with 300 nm of PbI_2 onto the substrate using a Lesker Mini Spectros evaporator to form an inorganic template. For textured tandems 40 nm of CsBr was coevaporated with 400 nm PbI_2. This inorganic template film is converted with an organohalide vapor in a close space sublimation configuration[1]. To complete the cell, 25 nm of C_{60} is thermally evaporated in a custom evaporator at a rate of 0.3 Å/s. Next, 225 cycles of atomic layer deposited SnO_x is deposited using a PICOSUN R-200 Standard ALD system (95 degrees, 0.12 nm/cycle). 35 nm of IZrO with sheet resistance around 250 ohm/square is RF sputtered using a Leybold Univex coating system from a 4-inch 98% In_2O_3 +2% ZrO target with deposition power of 70W and working pressure 2.7 µbar with ~0.14% of O_2 to Ar ratio through a shadow mask. The top silver grid (300 nm, 1 Å/s) is evaporated through a shadow mask. 110 nm MgF_x is evaporated as an antireflection layer through a shadow mask (140 nm for textured tandems).

Passivation treatments: where passivation of the perovskite is noted, 100 µl of a solution of 0.5 mg/ml $PDAI_2$ in IPA or 0.1 mg/ml PCl + 0.5 mg/ml 4F-PEACl was dynamically spin coated on the perovskite film at 5000 RPM and spun for 30s before 5 minutes of annealing at 100°C. For tandems on textured silicon evaporated $EDAI_2$ passivation[6] (3 nm) was used without further post treatment.

Bottom cell fabrication: The Si bottom cells were fabricated using 190 mm-thick, shiny-etched n-type float-zone monocrystalline Si wafers. After covering the front side of the wafers with a plasma enhanced chemical vapor deposition (PECVD)-grown SiN_x layer, a random pyramid texture (average peak height 0.9 µm,, max height 2 µm[7]) was formed at the rear side using a potassium hydroxide solution. The SiN_x at the front side was removed after the texturization. For fully textured wafers the SiN_x layer was omitted so both front and back were textured. Next the wafers received a wet-cleaning procedure. Prior to the PECVD processes, the wafers were dipped in a hydrofluoric acid solution to remove the thin chemical oxide at the surface. The hydrogenated amorphous and nanocrystalline Si layers were deposited by a plasma enhanced chemical vapor deposition (PECVD) system. On the front side, a-Si:H(i), a-Si:H(n), nc-Si:H(n) and nc-SiO_x:H(n) were deposited at 200 ºC. On the rear side, the a-Si:H(i) deposition at 200 ºC was followed by the depositions of ultra-thin SiO_x and nc-Si:H(p) layers at 175 ºC. Next, a 40 nm-thick ITO was deposited through a 1.1 × 1.1 cm^2 shadow mask on the rear side, then a 1 wt% SiO_2-NP dispersion was spin coated to fill the pyramid valleys to leave about 15% of the pyramid surface exposed for contacting with a 150 nm-thick sputtered Ag layer. The cells then received a full-area, 500 nm-thick SiO_x by PECVD, and an additional 150 nm-thick Ag to enhance their durability during perovskite processing. The front side of all cells were completed with a 20 nm-thick ITO sputtered through shadow masks with openings of 1.1 × 1.1 cm^2. The 4" wafers were then pre-scribed into 2.5 x 2.5 cm^2 substrates using a TRUMPF laser system operating at a wavelength of 1064 nm and subsequently cleaved manually along the scribed lines by hand. The wafers were then annealed at 210 °C for 30 min to recover sputter damage at which point they were ready for perovskite top cell processing.

A picture of representative solar cells (left: single junction perovskite, middle flat tandem, right textured tandem) (a)front and b) back) is shown below. The SJ device pictured uses Cu electrode, but Ag was used

for the devices in this work. In our experience the electrode metal choice does not affect performance for these devices. Some damage to the silver back contact from the organohalide vapor reaction is observed in the flat tandem, but >82% FF was still achieved. On the textured sample this was prevented by covering the back with a clean glass substrate to prevent vapor contact with the silver.

a)

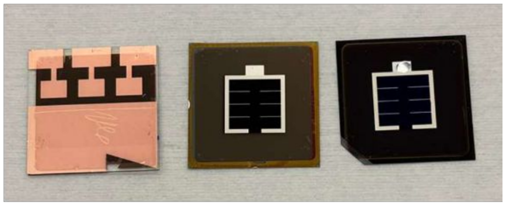

b)

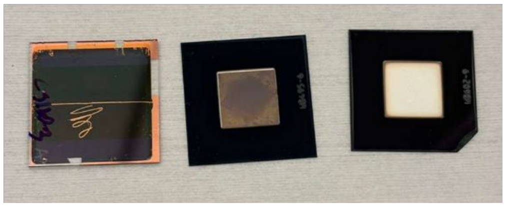

Characterization details

UV-vis measurements were performed with a PerkinElmer Lambda 900 UV-Vis-NIR spectrophotometer fitted with an integrating sphere.

Photoluminescence quantum yield and photoluminescence spectra were measured with a homemade system. A 532 nm laser is input into an integrating sphere with a fiber optic cable. The laser was calibrated with a photodetector to deliver the same photon flux as would be absorbed under 1 sun light for a bandgap of 1.60 eV. The laser shines onto the sample. An Ocean Optics USB2000 detector and Ocean Insight Maya2000 Pro detector collect sample emission and laser excitation intensities, respectively, using a bifurcated fiber from ThorLabs. As a calibration check, three fluorescent test samples with high specified PLQY (≈70%) supplied from Hamamatsu Photonics were measured where the specified value could be accurately reproduced within a small relative error of less than 5%. For each measurement run a dark calibration (laser blocked by the shutter, integrating sphere closed) and a light calibration (laser incident to integrating sphere with no sample in the sphere) was taken to understand the noise floor and to calibrate the laser absorbance. The typical integration times were 1s for the Maya2000 Pro and 10 ms for the USB2000 detector.

X-ray diffraction was performed in Bragg Brentano geometry with a copper Kα source with a Kβ filter using a PANalytical Xpert Pro MPD.

For the *JV* measurements under simulated sunlight, a solar simulator (WACOM) equipped with intensity-adjustable Xe and halogen Ha lamps was used. These measurements were performed prior to the EQE measurements, and at pre-set lamp intensities adjusted using filtered reference silicon (Si) cells (WPVS) with certificate numbers 41401-PTB-09 and 41402-PTB-08. Following the subsequent EQE measurements, the measurement artifacts were corrected according to Steiner et al.[8], and the mismatch and *Z*-factors corresponding the *JV* measurements were calculated as M_{top} = 1.013, M_{bot} = 0.973, Z_{top} = 0.987 and Z_{bot} = 0.972. The *JV* measurements were not repeated by recalibrating the lamp intensities accounting for these factors. For single junction solar cells, shadow masks are used to define the illuminated solar cell area to 0.1 cm^2. The measurements are done in ambient air without cooling. For tandem solar cells, a shadow mask is used to define the illuminated solar cell area to 1 cm^2 and the cell is actively cooled to 25 °C.

Mismatch measurements used to understand J_{sc}/FF tradeoffs upon current matching[9] were performed on the same calibrated WACOM solar simulator under simulated one sun light. To add bias light to a specific subcell, additional light is supplied using a custom setup from either blue LEDs (428 nm, contributing only to top cell current) or infrared LEDs (940 nm, contributing only to bottom cell current). Prior to measurements under simulated sunlight, the LED drive-currents were calibrated to the induced J_{sc} values in the subcells under dark. This was accomplished by setting one of the LEDs to near their maximum powers, to induce about 4 mA/cm^2, then increasing the intensity of the other LED step-by-step, while monitoring and recording the measured device J_{sc} and then repeating for the opposite LED configuration. For both LEDs, the measured J_{sc} was < 0.1 mA/cm^2 when only one of them was turned on and set to its maximum (~4 mA/cm^2), indicating an insignificant influence of shunting or luminescent coupling on device current. For the measurements shown in Figure S9, one LED was turned on at a time. The parameter 'Bias light' in the x-axis corresponds to the LED-induced additional J_{sc} in a subcell between ± 3 mA/cm^2 and is positive when the blue LED is used, and negative when the IR LED is used. In this case, when the LEDs are used under simulated sunlight, the measured device J_{sc} converges to the J_{sc} of the subcell limiting the devices for any given bias.

The external quantum efficiency of perovskite solar cells was measured with a custom-made spectral response set-up where the samples were irradiated with chopped light at a frequency of 217 Hz and the response measured with a lock-in amplifier. For tandem cells, during top-cell measurement, the device is biased with 0.7 V and IR light, while during bottom-cell measurements, the device is biased with 1.1 V and blue light.

SEM images were taken using a Zeiss Gemini 2 microscope using a secondary electron detector and an acceleration voltage of 3 kV.

Photoluminescence-Based iV_{OC} Imaging Fraunhofer ISE

A commercial measurement system developed at Fraunhofer ISE and manufactured by Intego GmbH was used for photoluminescence (PL) imaging. The system is equipped with a 450 nm laser to excite the perovskite top cell and an 808 nm laser to excite the silicon bottom solar cell. Optical filters were used to subcell selectively capture the PL signal of either subcell in a silicon charge-coupled device (CCD) camera. The illumination intensities were adjusted to the AM1.5G equivalent photon fluxes, using the relative EQEs and following the approach described by Meusel et al.[10]. The PL images were converted to iV_{OC} images using a calibration procedure described by Fischer et al.[11]. The absorptance of the investigated solar cells was approximated from the EQE.

JV, EQE Fraunhofer ISE

EQE measurements were done with an in-house measurement setup. A Xenon lamp is used as light source. The light is chopped at ~133 Hz and directed through a double grating monochromator to generate monochromatic illumination. A transimpedance amplifier provides bias voltage during the measurements and amplifies the signal, which is subsequently detected by a lock-in amplifier. For selective excitation, infrared (940 nm) and blue (460 nm) LEDs were used for the measurement of the perovskite and silicon sub cells, respectively. A bias voltage was applied to the device for each sub-cell measurement, following the international standard procedure[12]. The temperature was maintained at 25 °C.

A Wavelabs SINUS 220 light-emitting diode (LED)-based solar simulator containing 20 different LED channels were used for the JV measurements. The LEDs intensity were adjusted to ensure that the spectrum is correctly adjusted such that the photocurrent generated by each sub cell under the solar simulator spectrum matches the photocurrent generated under the AM1.5g spectrum[10,12]. For this, the spectral responses (SR) of the sub cells were measured prior to the JV measurement. The spectrum was then calculated using the relative SRs according to the procedure described by Chojniak et al[13]. The measurement

was done from -0.05 V to 1.9 V with a voltage step of 0.004 V and a scan rate of 0.1 mV/s first in reverse, then in forward scan directions. The temperature of the measurement chuck was maintained at 25 °C.

Supplementary Figures and Tables:

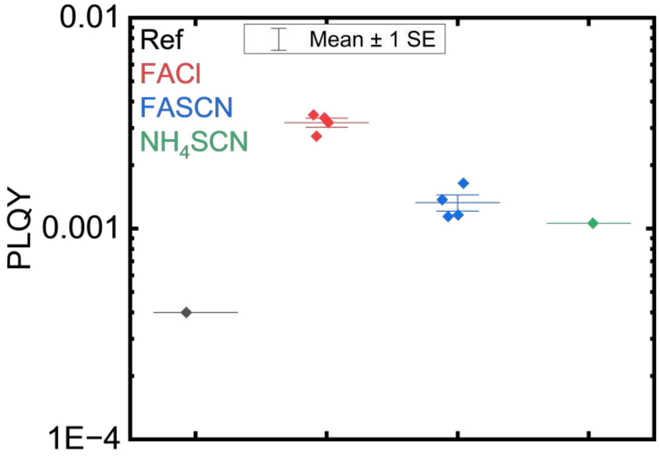

Figure S1. PLQY of PVD-CSS films with different additives. FACl shows the highest PLQY so it was chosen for the rest of the work.

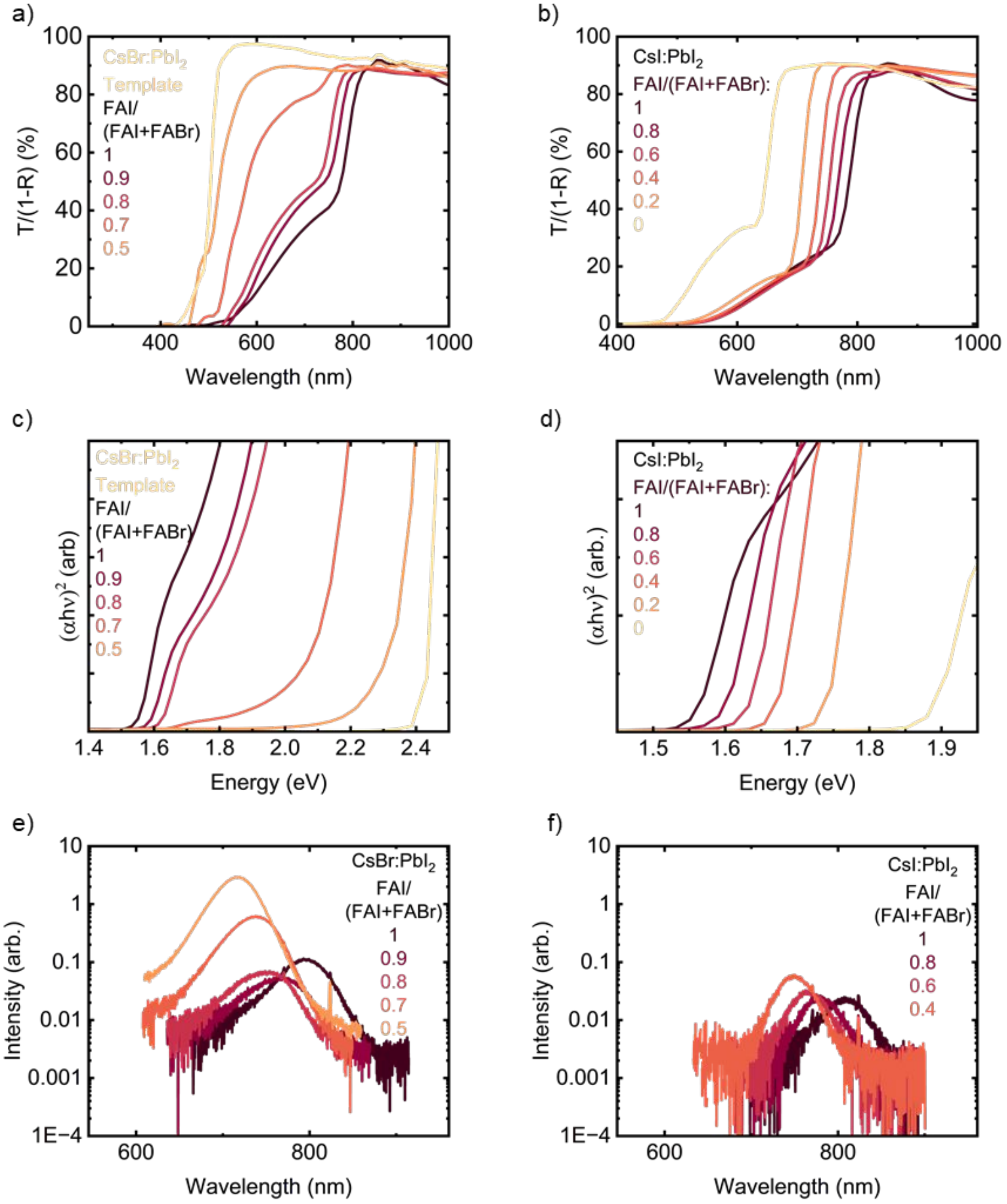

Figure S2. Reflection corrected transmission, Tauc plots, and PL spectra for films using a), c), d) 20 nm CsBr: 200 nmPbI$_2$ templates vs b), d), e) 30 nm CsI: 300 nm PbI$_2$ templates. The CsI:PbI$_2$ templates were thickened only to achieve higher short circuit current densities. As bromine content increases, the absorption feature starts to show less absorption past the band edge with multiple absorption features and an increased and broadened PL, which may indicate the start of high/low bandgap phase splitting and the need to manage these phenomena in this process to achieve bandgaps of >1.70 eV. However, good tunability especially within the tandem-relevant band gap range is clear. When referenced in the main text or plots, the bandgap from Tauc plots is used.

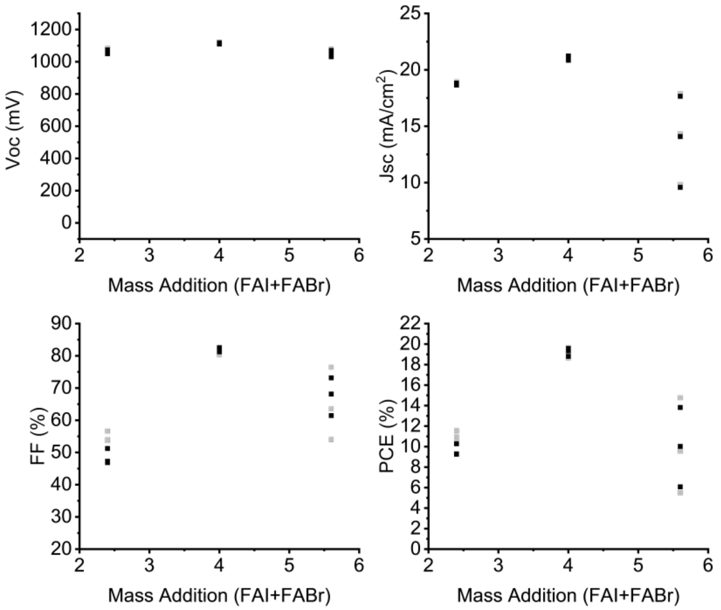

Figure S3. The performance trend of solar cells with 0.8 FAI/(FAI+FABr) over various FAI+FABr mass additions showing an optimum FAI+FABr mass addition for the reaction of 4 mg.

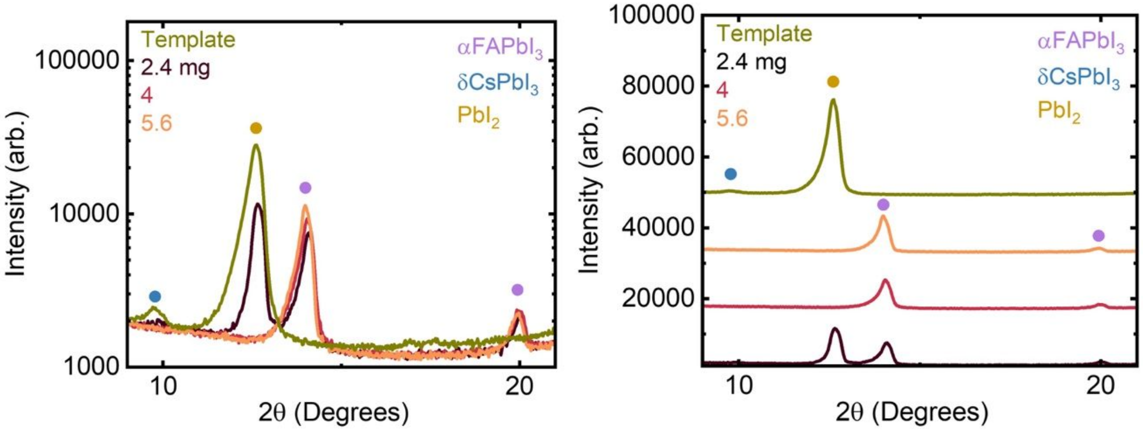

Figure S4. The XRD of the complete devices shown in Figure S3 showing that perovskites without delta phase or PbI_2 impurities can be fabricated, enabling the champion single junction device to achieve >20% PCE.

Table S1. The JV parameters of the devices shown in Figure 1b and 1d. The prescript "f" denotes forward scan and "r" denotes reverse scan.

Condition	E_g (eV)	fV_{oc} (mV)	rV_{oc} (mV)	fJ_{sc} (mA/cm^2)	rJ_{sc} (mA/cm^2)	fFF (%)	rFF (%)	fPCE (%)	rPCE (%)
1.0	1.55	1015	1018	22.2	22.1	74.2	77.7	16.6	17.4
0.8	1.60	1064	1072	21.7	21.7	78.5	79.1	18.1	18.4
0.6	1.63	1071	1070	21.2	21.2	81.1	81.4	18.4	18.5
0.8 PDAI	1.60	1098	1099	21.7	21.6	81.0	81.7	19.3	19.4
0.8 Bimoelcular	1.60	1126	1131	21.3	21.3	83.5	83.4	20.1	20.1

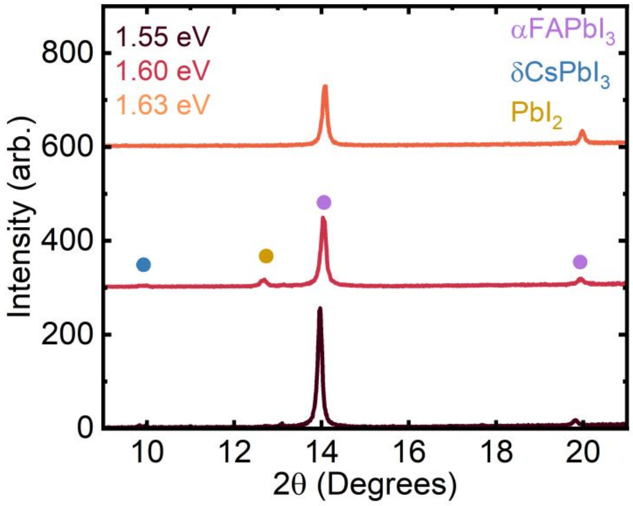

Figure S5. X-ray diffraction of the thin films whose JV performance is reported in Figure 1b and EQE Figure 1c. We note that EQE absorption features between 400 nm and 600 nm correlate to the presence of some PbI_2 and delta $CsPbI_3$- type phases. These phases often arise after prolonged air exposure. These films were exposed to the uncontrolled ambient atmosphere for several hours (temperature ~20°C, humidity ~60% during fabrication in August) during the PL and UV-Vis measurements presented above, as well as the CSS fabrication process. Further refinement of the reaction conditions and control of ambient air exposure reduces these side phase incorporations. Figure S3 shows the same 1.60 eV perovskite recipe without delta phase formation due to air exposure, as these films were exposed to air for the minimum time possible through the fabrication process (~1 hour).

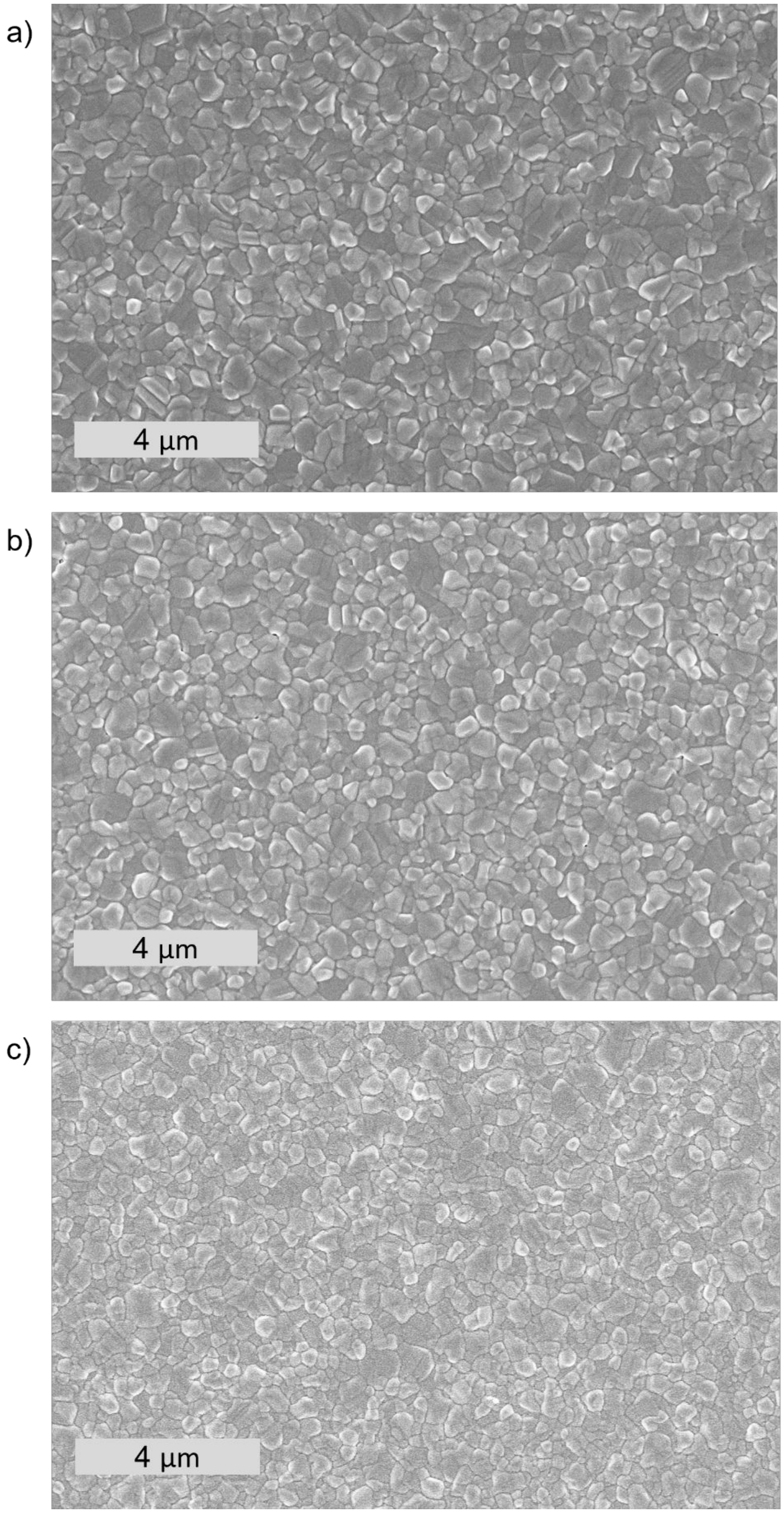

Figure S6. Top view SEM of PVD-CSS films with an FAI/(FAI+FABr) ratio of a) 1 b) 0.8 and c) 0.6

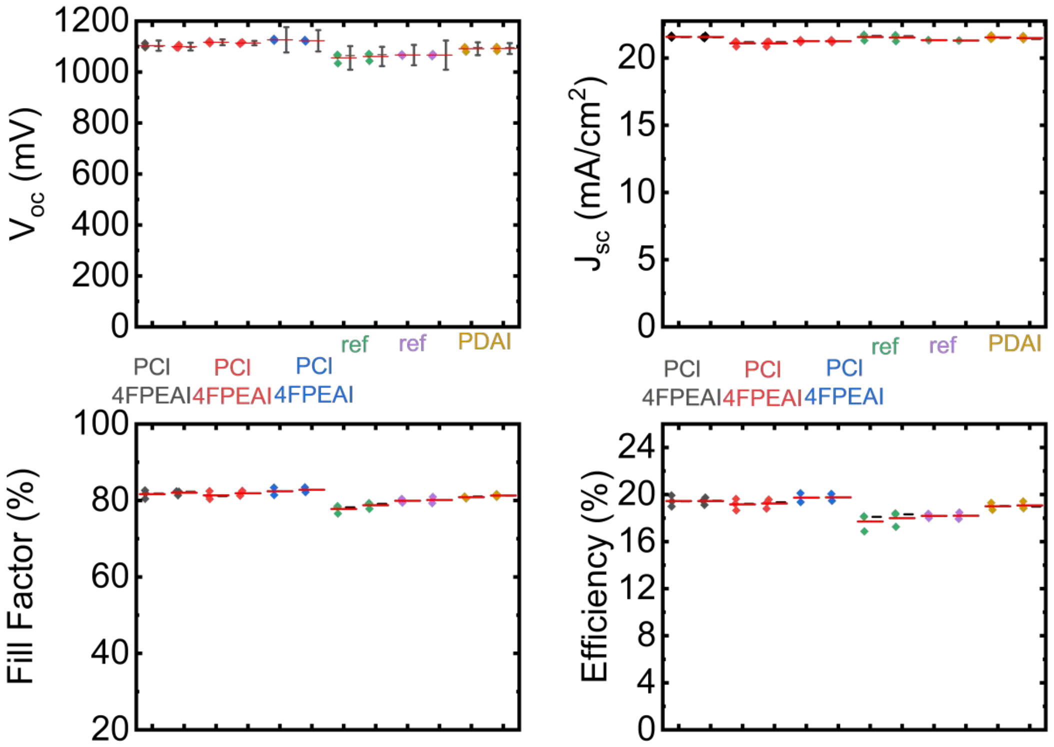

Figure S7. Single-junction solar cells using the optimized process with 0.8 FAI/(FAI+FABr) by mass and 4 mg FAI+FABr mass addition, with and without surface passivation showing the robustness of the process. Shunted cells removed from the analysis.

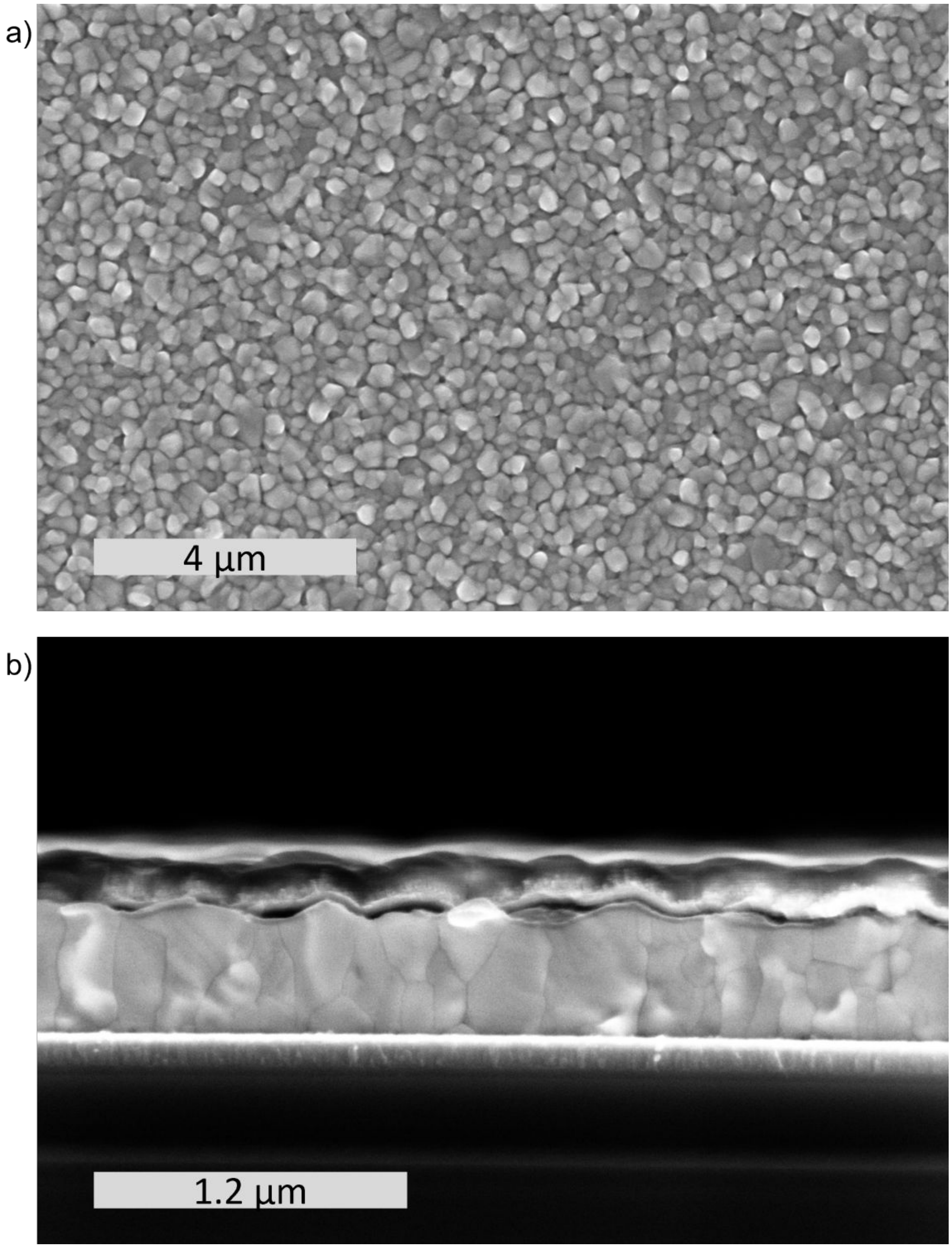

Figure S8 a) top view and b) cross-section SEM of a PVD-CSS tandem device on flat silicon.

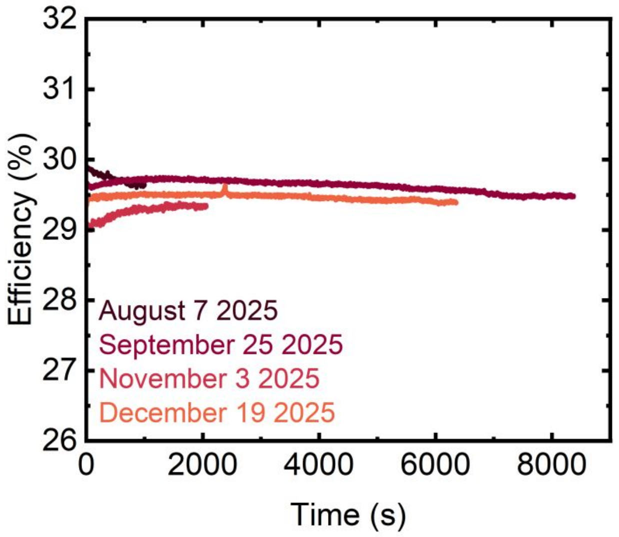

Figure S9. Maximum power point tracking unencapsulated in air at 25°C for the champion tandem cell on August 7 for 1000 seconds and September 25 for >8000 seconds. Minor losses are seen for operational periods at 25C in air unencapsulated for >2 hours (e.g. September 25).

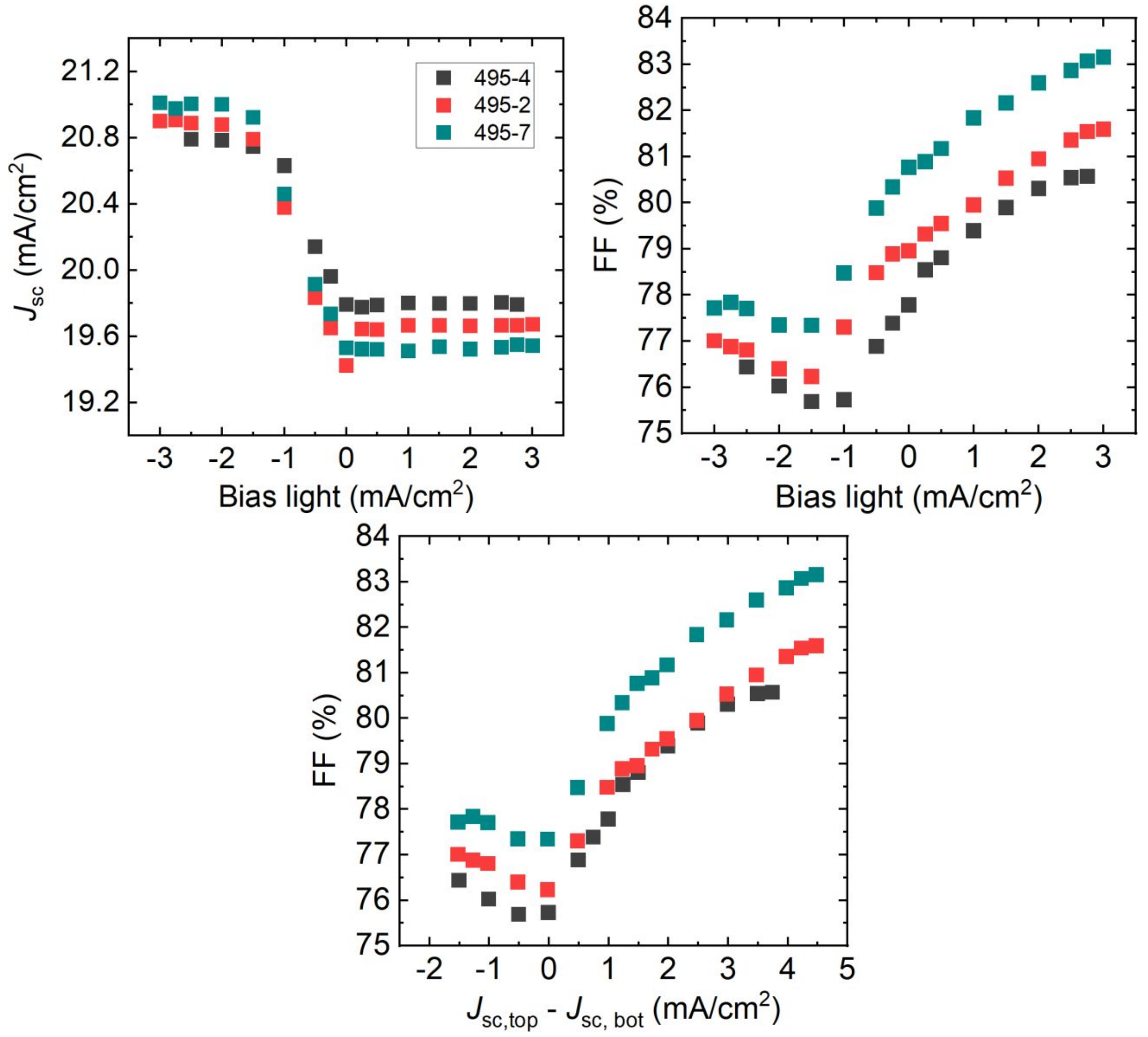

Figure S10. Mismatch analysis of a few average-performing perovskite-silicon tandem solar cells Applying no light bias or a positive (i.e., blue LED) bias yields a nearly constant $J_{sc} \approx J_{sc,bot,0}$ (i.e., about 19.7 mA/cm^2), showing that the bottom cell limits the current. When the IR LED is boosted to make the top cell current-limiting, e.g., at -3 mA/cm^2, the measured current density increases then plateaus at $J_{sc} \approx J_{sc,top,0}$ (i.e., about 20.9 mA/cm^2). These values were then used to construct the FF versus $J_{sc,top} - J_{sc,bot} = b + J_{sc,top,0} - J_{sc,bot,0}$, illustrated in the bottom figure. Overall, the J_{sc} trend shows that the cell is limited by the current output from the bottom cell and the top cell is capable of outputting approximately 1 mA/cm^2 more than the filtered bottom cell. The FF trend shows a FF minimum at current matched conditions[13] and preliminarily indicate that the bottom cell limited FF is 3% FF higher than the top cell limited FF. This shows that slight bottom limitation can be beneficial. In this case, going to a more current matched condition would decrease the FF of the cells, and compensate for a portion of the J_{sc} gain. Thus, this bandgap appears to be an approximately optimal choice for these devices as-is. Additional improvements to the top cell FF (from improved pseudoFF, shunt resistance, series resistance, etc.) would likely allow the realization of gains from better current matching.

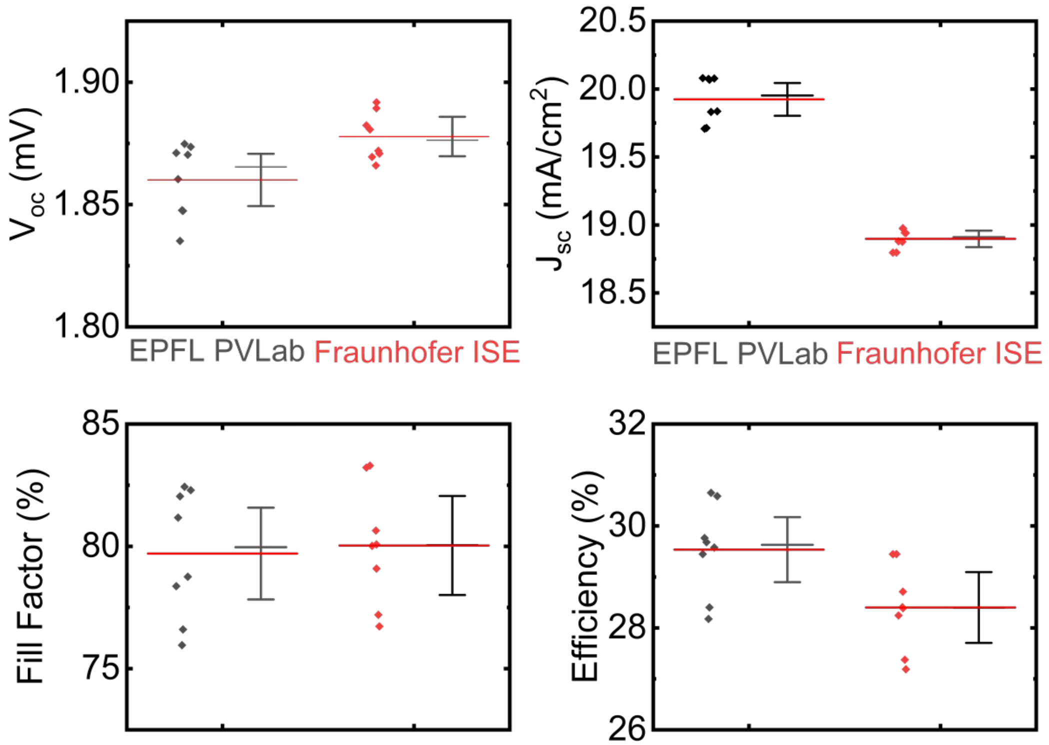

Figure S11. The four selected tandem cells measured at EPFL PVLab vs Fraunhofer ISE. Both forward and reverse scans are included but not differentiated. At EPFL PVLab, the J_{sc} is higher while V_{oc} and FF are marginally lower. In the context of the mismatch measurements shown in figure S7, this suggests a spectral difference changing the current output of the limiting bottom cell. We note that the requirement of precise spectral matching makes high precision measurements of multijunction solar cells difficult[13], an aspect that deserves increased attention in the tandem community. This is why measurements at multiple institute or measurements at calibration labs is of high importance. The preconditioning states are also different, as at EPFL a shutter is utilized that opens shortly before the measurement while at Fraunhofer the cell is continually exposed to V_{oc} illumination before the JV sweep.

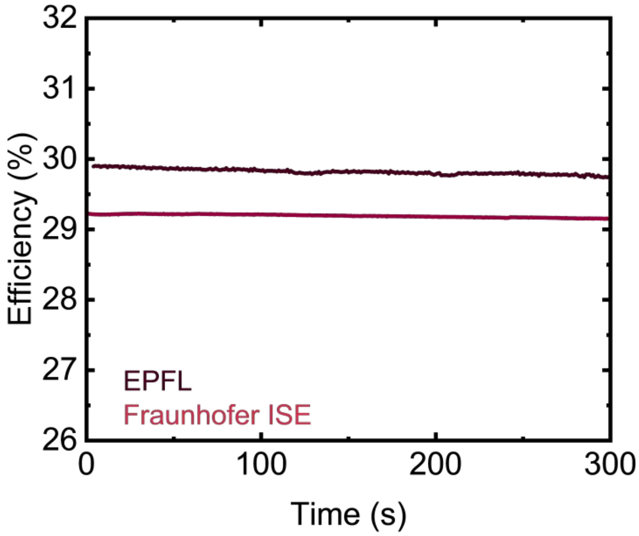

Figure S12. Maximum power point tracking at EPFL vs fixed voltage tracking three weeks later at Fraunhofer ISE giving stable power conversion efficiencies >29%. The lower MPPT is likely caused by the different spectral settings as described in chapter "characterization details" and not predominantly due to degradation, as shown by subsequent MPPT tracking of the device achieving nearly the same PCE (Figure S4).

Table S2. Champion and mean JV parameters from the reverse scan for the four selected tandem cells measured at EPFL and Fraunhofer ISE.

	V_{oc} (mV)	J_{sc} (mA/cm^2)	FF (%)	PCE (%)
Champion EPFL	1875	19.8	82.4	30.7
Batch mean EPFL	1860	19.9	79.7	29.5
Champion ISE	1881	18.8	83.3	29.4
Batch mean ISE	1878	18.9	80.0	28.4

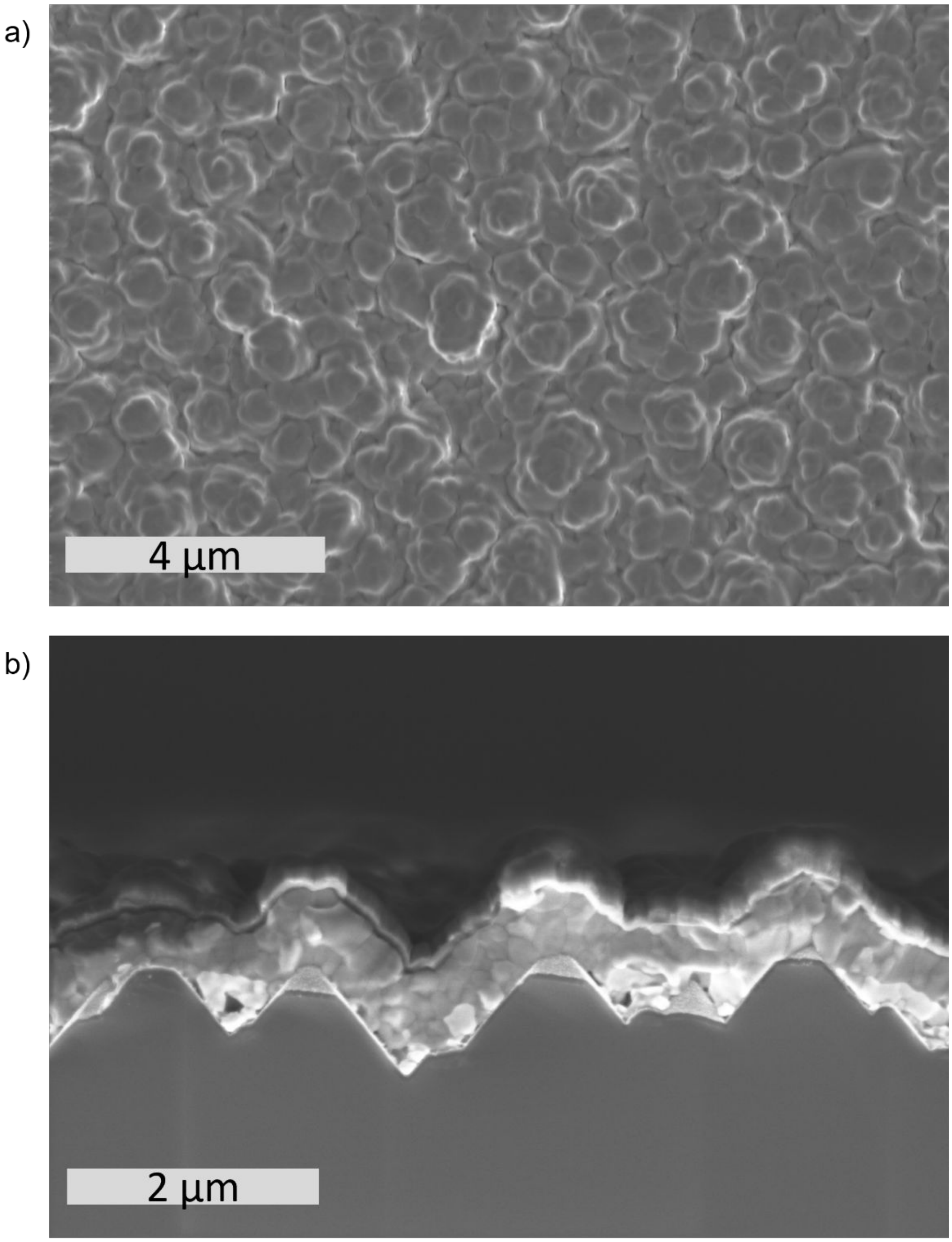

Figure S13 a) top view and b) cross-section image of a CSS tandem on textures using FAI+FABr 6.4 mg with FAI/(FAI+FABr) =0.8. In the cross-section image, visible regions of under-converted template appear to exist in the valleys but the pyramid coverage appears to be excellent. Obvious shunting features were not found by SEM but must be remedied in future work to achieve >30% efficiency. We suspect that engineering substrate wetting and increasing template thickness will help decrease the likelihood of solid state dewetting[42].

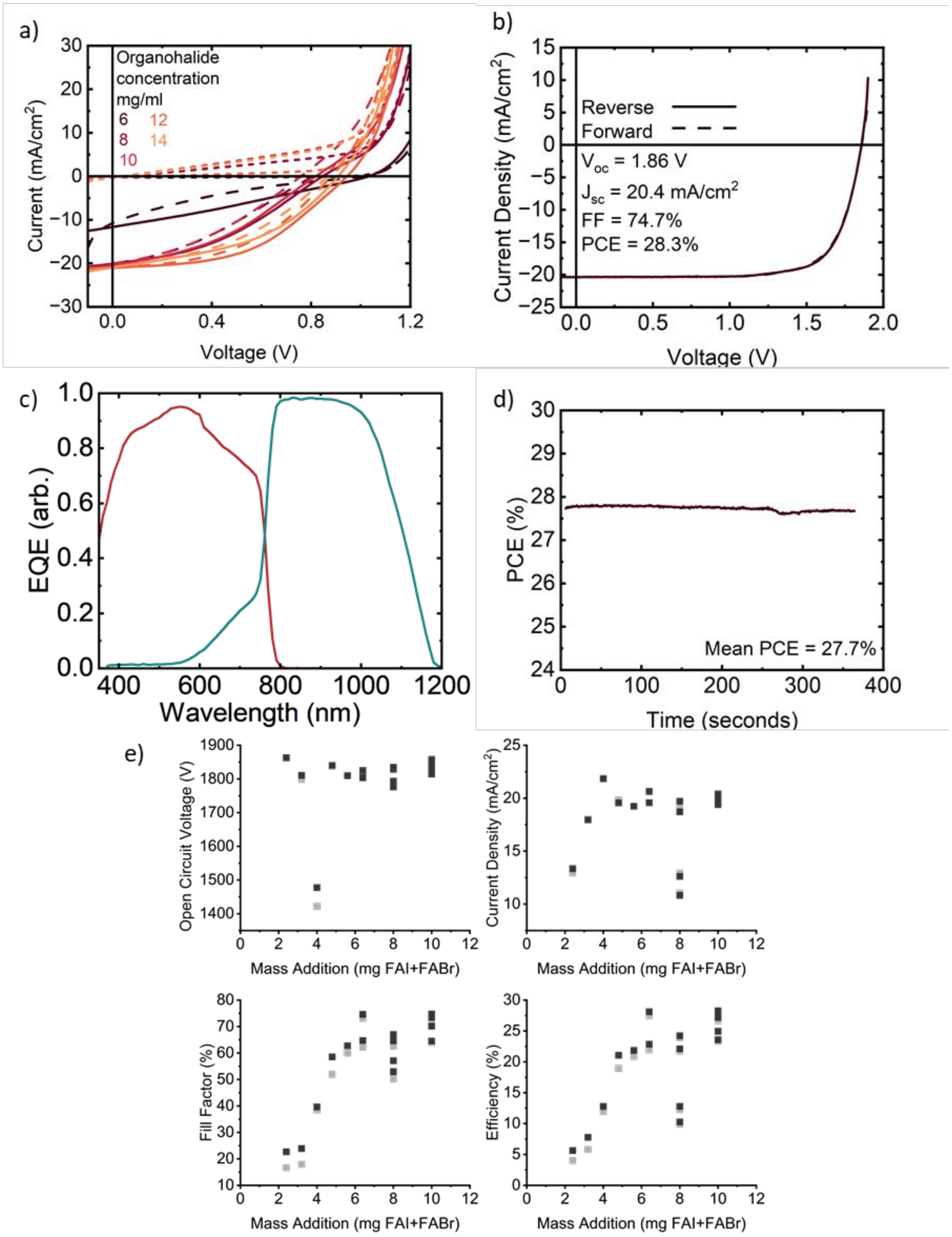

Figure S14. a) single junction perovskite solar cells on ohmic textured silicon substrates. The dark curves have notably low shunt resistance. b) JV and c) EQE and d) MPPT tracking of the champion perovskite-silicon tandem solar cell using a PVD-CSS absorber and a textured silicon bottom cell. e) organohalide FA+FABr mass addition optimization of PVD-CSS tandems on textured silicon.

References

(1) Guesnay, Q.; Sahli, F.; Artuk, K.; Turkay, D.; Kuba, A. G.; Mrkyvkova, N.; Vegso, K.; Siffalovic, P.; Schreiber, F.; Lai, H.; Fu, F.; Ledinský, M.; Fürst, N.; Schafflützel, A.; Bucher, C.; Jeangros, Q.; Ballif, C.; Wolff, C. M. Pizza Oven Processing of Organohalide Perovskites (POPOP): A Simple, Versatile and Efficient Vapor Deposition Method. *Adv. Energy Mater.* **2024**, *14* (10), 2303423. https://doi.org/10.1002/aenm.202303423.

(2) Li, G.; Ho, J. Y. L.; Wong, M.; Kwok, H. S. Reversible Anion Exchange Reaction in Solid Halide Perovskites and Its Implication in Photovoltaics. *J. Phys. Chem. C* **2015**, *119* (48), 26883–26888. https://doi.org/10.1021/acs.jpcc.5b09300.

(3) Xiong, W.; Hu, S.; Song, Y.; Dou, Y.; Liang, J.; Yuan, Z.; Deng, X.; Liu, M.; Liu, M.; Qiang, Z.; Ku, Z. Achieving High-Quality Wide Bandgap Perovskite Thin Films via Regulating the Halide Ion Exchange Order in Vapor-Solid Reaction. *Sol. RRL* **2025**, *9* (9), 2500053. https://doi.org/10.1002/solr.202500053.

(4) Sahli, F.; Miaz, N.; Salsi, N.; Bucher, C.; Schafflützel, A.; Guesnay, Q.; Duchêne, L.; Niesen, B.; Ballif, C.; Jeangros, Q. Vapor Transport Deposition of Methylammonium Iodide for Perovskite Solar Cells. *ACS Appl. Energy Mater.* **2021**, *4* (5), 4333–4343. https://doi.org/10.1021/acsaem.0c02999.

(5) Turkay, D.; Artuk, K.; Chin, X.-Y.; Jacobs, D. A.; Moon, S.-J.; Walter, A.; Mensi, M.; Andreatta, G.; Blondiaux, N.; Lai, H.; Fu, F.; Boccard, M.; Jeangros, Q.; Wolff, C. M.; Ballif, C. Synergetic Substrate and Additive Engineering for over 30%-Efficient Perovskite-Si Tandem Solar Cells. *Joule* **2024**, *8* (6), 1735–1753. https://doi.org/10.1016/j.joule.2024.04.015.

(6) Hu, S.; Pascual, J.; Liu, W.; Funasaki, T.; Truong, M. A.; Hira, S.; Hashimoto, R.; Morishita, T.; Nakano, K.; Tajima, K.; Murdey, R.; Nakamura, T.; Wakamiya, A. A Universal Surface Treatment for P-i-n Perovskite Solar Cells. *ACS Appl. Mater. Interfaces* **2022**. https://doi.org/10.1021/acsami.2c15989.

(7) Turkay, D.; Artuk, K.; Othman, M.; Sahli, F.; Champault, L.; Allebé, C.; Hessler-Wyser, A.; Jeangros, Q.; Ballif, C.; Wolff, C. M. Beyond Flat: Undulated Perovskite Solar Cells on Microscale Si Pyramids by Solution Processing. *ACS Energy Lett.* **2025**, *10* (3), 1397–1403. https://doi.org/10.1021/acsenergylett.5c00221.

(8) Steiner, M. A.; Geisz, J. F.; Moriarty, T. E.; France, R. M.; McMahon, W. E.; Olson, J. M.; Kurtz, S. R.; Friedman, D. J. Measuring IV Curves and Subcell Photocurrents in the Presence of Luminescent Coupling. *IEEE J. Photovolt.* **2013**, *3* (2), 879–887. https://doi.org/10.1109/JPHOTOV.2012.2228298.

(9) Köhnen, E.; Jošt, M.; Morales-Vilches, A. B.; Tockhorn, P.; Al-Ashouri, A.; Macco, B.; Kegelmann, L.; Korte, L.; Rech, B.; Schlatmann, R.; Stannowski, B.; Albrecht, S. Highly Efficient Monolithic Perovskite Silicon Tandem Solar Cells: Analyzing the Influence of Current Mismatch on Device Performance. *Sustain. Energy Fuels* **2019**, *3* (8), 1995–2005. https://doi.org/10.1039/C9SE00120D.

(10) Meusel, M.; Adelhelm, R.; Dimroth, F.; Bett, A. W.; Warta, W. Spectral Mismatch Correction and Spectrometric Characterization of Monolithic III–V Multi-junction Solar Cells. *Prog. Photovolt. Res. Appl.* **2002**, *10* (4), 243–255. https://doi.org/10.1002/pip.407.

(11) Fischer, O.; Bui, A. D.; Schindler, F.; Macdonald, D.; Glunz, S. W.; Nguyen, H. T.; Schubert, M. C. Versatile Implied Open-circuit Voltage Imaging Method and Its Application in Monolithic Tandem Solar Cells. *Prog. Photovolt. Res. Appl.* **2025**, *33* (1), 40–53. https://doi.org/10.1002/pip.3754.

(12) Photovoltaic Devices - Part 8-1: Measurement of Spectral Responsivity of Multi-Junction Photovoltaic (PV) Devices, 2017.

(13) Chojniak, D.; Schachtner, M.; Reichmuth, S. K.; Bett, A. J.; Rauer, M.; Hohl-Ebinger, J.; Schmid, A.; Siefer, G.; Glunz, S. W. A Precise Method for the Spectral Adjustment of LED and Multi-light Source Solar Simulators. *Prog. Photovolt. Res. Appl.* **2024**, *32* (6), 372–389. https://doi.org/10.1002/pip.3776.

(14) Li, G.; Ho, J. Y. L.; Wong, M.; Kwok, H. Low Cost, High Throughput and Centimeter-scale Fabrication of Efficient Hybrid Perovskite Solar Cells by Closed Space Vapor Transport. *Phys. Status Solidi RRL – Rapid Res. Lett.* **2016**, *10* (2), 153–157. https://doi.org/10.1002/pssr.201510386.

(15) Guo, Q.; Li, C.; Qiao, W.; Ma, S.; Wang, F.; Zhang, B.; Hu, L.; Dai, S.; Tan, Z. The Growth of a $CH_3NH_3PbI_3$ Thin Film Using Simplified Close Space Sublimation for Efficient and Large Dimensional Perovskite Solar Cells. *Energy Environ. Sci.* **2016**, *9* (4), 1486–1494. https://doi.org/10.1039/C5EE03620H.

(16) Gu, L.; Wang, S.; Fang, X.; Liu, D.; Xu, Y.; Yuan, N.; Ding, J. High-Performance Large-Area Perovskite Solar Cells Enabled by Confined Space Sublimation. *ACS Appl. Mater. Interfaces* **2020**, *12* (30), 33870–33878. https://doi.org/10.1021/acsami.0c10830.

(17) Zhu, X.; Yang, D.; Yang, R.; Yang, B.; Yang, Z.; Ren, X.; Zhang, J.; Niu, J.; Feng, J.; Liu, S. (Frank). Superior Stability for Perovskite Solar Cells with 20% Efficiency Using Vacuum Co-Evaporation. *Nanoscale* **2017**, *9* (34), 12316–12323. https://doi.org/10.1039/C7NR04501H.

(18) Luo, L.; Zhang, Y.; Chai, N.; Deng, X.; Zhong, J.; Huang, F.; Peng, Y.; Ku, Z.; Cheng, Y.-B. Large-Area Perovskite Solar Cells with $Cs_x FA_{1-x} PbI_{3-y} Br_y$ Thin Films Deposited by a Vapor–Solid Reaction Method. *J. Mater. Chem. A* **2018**, *6* (42), 21143–21148. https://doi.org/10.1039/C8TA06557H.

(19) Harding, A. J.; Kuba, A. G.; McCandless, B. E.; Das, U. K.; Dobson, K. D.; Ogunnaike, B. A.; Shafarman, W. N. The Growth of Methylammonium Lead Iodide Perovskites by Close Space Vapor Transport. *RSC Adv.* **2020**, *10* (27), 16125–16131. https://doi.org/10.1039/D0RA01640C.

(20) Kuba, A. G.; Harding, A. J.; Richardson, R. J.; Mccandless, B. E.; Das, U. K.; Dobson, K. D.; Shafarman, W. N. Two-Step Close-Space Vapor Transport of MAPbI 3 Solar Cells : Effects of Electron Transport Layers and Residual PbI 2. **2022**. A*CS Appl. Energy Mater. 5, 9, 10731–10741* https://doi.org/10.1021/acsaem.2c01468.

(21) Kuba, A.; Du, B.; Harding, A.; Dobson, K.; McCandless, B.; Das, U.; Shafarman, W. The Role of Oxygen Exposure on the Performance of All-Vapor-Processed Perovskite Solar Cells with CuPC Hole Transport Layers; Institute of Electrical and Electronics Engineers Inc., 2021; pp 1451–1454. https://doi.org/10.1109/PVSC43889.2021.9518616.

(22) Kuba, A. G.; Santiwipharat, C.; Richardson, R. J.; Das, U. K.; Dobson, K. D.; Shafarman, W. N. Air-Induced Conductivity Loss in Fullerene ETLs Can Drive Charge Extraction Losses in Vapor-Deposited Perovskite Solar Cells. *ACS Appl. Energy Mater.* **2024**, *7* (24), 11921–11928. https://doi.org/10.1021/acsaem.4c02306.

(23) Zhang, G.; Luo, W.; Dai, H.; Li, N.; Li, Y.; Peng, Y.; Huang, F.; Ku, Z.; Cheng, Y.-B. Ultrafast Growth of High-Quality $Cs_{0.14} FA_{0.86} Pb(Br_x I_{1-x})_3$ Thin Films Achieved Using Super-Close-Space Sublimation. *ACS Appl. Energy Mater.* **2022**, *5* (5), 5797–5803. https://doi.org/10.1021/acsaem.2c00132.

(24) Tie, F.; Duan, C.; Hu, S.; Dou, Y.; Tan, Q.; Fan, J.; Lu, J.; Xu, M.; Ku, Z. Valine-Modified PbI_2 for the Growth of Pinhole-Free Lead Halide Perovskite Thin Films by Vapor–Solid Reaction. *ACS Appl. Energy Mater.* **2023**, *6* (12), 6681–6688. https://doi.org/10.1021/acsaem.3c00676.

(25) Liang, J.; Liu, M.; Hu, S.; Xiong, W.; Yuan, Z.; Qiang, Z.; Ku, Z. Regulating the Crystallization and Morphology of PbI_2 Precursor Films for the Growth of High-Quality Perovskite Films via Vapor–Solid Reaction. *ACS Appl. Mater. Interfaces* **2025**, *17* (12), 18535–18545. https://doi.org/10.1021/acsami.5c02229.

(26) Hu, S.; Duan, C.; Du, H.; Zeng, S.; Kong, A.; Chen, Y.; Peng, Y.; Cheng, Y.-B.; Ku, Z. A Stress Relaxation Strategy for Preparing High-Quality Organic–Inorganic Perovskite Thin Films *via* a Vapor–Solid Reaction. *J. Mater. Chem. A* **2023**, *11* (43), 23387–23396. https://doi.org/10.1039/D3TA04666D.

(27) Wang, Y.; Lv, P.; Pan, J.; Chen, J.; Liu, X.; Hu, M.; Wan, L.; Cao, K.; Liu, B.; Ku, Z.; Cheng, Y.; Lu, J. Grain Boundary Elimination via Recrystallization-Assisted Vapor Deposition for Efficient and Stable Perovskite Solar Cells and Modules. *Adv. Mater.* **2023**, *35* (44), 2304625. https://doi.org/10.1002/adma.202304625.

(28) Zhao, F.; Zhong, J.; Zhang, L.; Yong, P.; Lu, J.; Xu, M.; Cheng, Y.; Ku, Z. Two-Step Vapor-Solid Reaction for the Growth of High-Quality CsFA-Based Lead Halide Perovskite Thin Films. *Sol. RRL* **2023**, *7* (11), 2300062. https://doi.org/10.1002/solr.202300062.
(29) Fan, J.; Chen, Y.; Kong, A.; Tan, Q.; Zhong, J.; Zhang, L.; Peng, Y.; Liang, G.; Ku, Z. Fabrication of Large-Grained Perovskite Films Utilizing a Recrystallization Approach Involving Multiple Vapor Annealing Steps. *ACS Appl. Energy Mater.* **2024**, *7* (9), 3740–3749. https://doi.org/10.1021/acsaem.4c00080.
(30) Duan, C.; Dou, Y.; Hu, S.; Deng, X.; Liu, M.; Liu, M.; Liang, G.; Peng, Y.; Cheng, Y.-B.; Ku, Z. Stoichiometric Gradient Rebalancing Achieves Surface Reconstruction and Bulk Homogenization in High-Performance Vapor-Deposited Perovskite Solar Cells. *J. Mater. Chem. A* **2025**, *13* (30), 24675–24684. https://doi.org/10.1039/D5TA03102H.
(31) Duan, C.; Zhong, J.; Hu, S.; Dou, Y.; Lu, J.; Cheng, Y.; Ku, Z. Oriented Growth for Efficient and Scalable Perovskite Solar Cells by Vapor–Solid Reaction. *Adv. Funct. Mater.* **2024**, *34* (21), 2313435. https://doi.org/10.1002/adfm.202313435.
(32) Dou, Y.; Lv, P.; Yuan, Z.; Xiong, W.; Liang, J.; Peng, Y.; Liang, G.; Ku, Z. Enhanced Buried Interface Engineering for Efficient Inverted Perovskite Solar Cells Fabricated via Vapor–Solid Reaction. *Small Methods* **2025**, *9* (4), 2401339. https://doi.org/10.1002/smtd.202401339.
(33) Hu, S.; Zeng, S.; Deng, X.; Hou, P.; Du, H.; Dou, Y.; Xiong, W.; Pan, J.; Peng, Y.; Cheng, Y.-B.; Ku, Z. Scalable Impregnation Method for Preparing a Self-Assembled Monolayer in High-Performance Vapor-Deposited Lead-Halide Perovskite Solar Cells. *ACS Nano* **2025**, *19* (15), 15018–15029. https://doi.org/10.1021/acsnano.5c01479.
(34) Hu, S.; Hou, P.; Duan, C.; Zeng, S.; Dou, Y.; Deng, X.; Zhang, Y.; Lu, J.; Cheng, Y.-B.; Peng, Y.; Ku, Z. Unveiling the Role of Cs Ion in Perovskite Phase Formation during Solid–Vapor Reactions. *Chem. Eng. J.* **2024**, *499*, 156259. https://doi.org/10.1016/j.cej.2024.156259.
(35) Wang, Y.; Chen, J.; Zhang, Y.; Lv, P.; Pan, J.; Hu, M.; Tan, W. L.; Ku, Z.; Cheng, Y.; Simonov, A. N.; Lu, J. Scalable Fabrication of High-Performance Perovskite Solar Cell Modules by Mediated Vapor Deposition. *Adv. Mater.* **2024**, *36* (49), 2412021. https://doi.org/10.1002/adma.202412021.
(36) Zhang, L.; Zhong, J.; Kong, A.; Chen, Y.; Fan, J.; Tan, Q.; Peng, Y.; Liang, G.; Ku, Z. A Layering Technique for Achieving Pinhole-Free Organic–Inorganic Halide Perovskite Thin Films through the Vapor–Solid Reaction. *Sustain. Energy Fuels* **2024**, *8* (11), 2485–2493. https://doi.org/10.1039/D4SE00320A.
(37) Rodkey, N.; Gomar-Fernández, I.; Ventosinos, F.; Roldan-Carmona, C.; Koster, L. J. A.; Bolink, H. J. Close-Space Sublimation as a Scalable Method for Perovskite Solar Cells. *ACS Energy Lett.* **2024**, *9* (3), 927–933. https://doi.org/10.1021/acsenergylett.3c02794.
(38) Gomar-Fernández, I.; Gil-Escrig, L.; Rodkey, N.; Ventosinos, F.; Senno, M.; Roldán-Carmona, C.; Held, V.; Sessolo, M.; Bolink, H. J. Large-Area Close-Space Sublimation Enables the Fabrication of Efficient and Stable Perovskite Solar Cells. *EES Sol.* **2025**, *1* (6), 1126–1134. https://doi.org/10.1039/D5EL00145E.
(39) Zhang, Y.; Zhu, Y.; Sun, J.; Hu, M.; Chen, J.; Duan, B.; Hu, S.; Hou, P.; Tan, W. L.; Ku, Z.; Yang, W.; Lu, J. Low Pressure Chemical Vapor Deposited Perovskite Enables All Vacuum-Processed Monolithic Perovskite-Silicon Tandem Solar Cells. *Adv. Energy Mater.* **2025**, *15* (27), 2405377. https://doi.org/10.1002/aenm.202405377.
(40) Kuba, A. G.; Du, B.; Harding, A. J.; Dobson, K. D.; McCandless, B. E.; Das, U. K.; Shafarman, W. N. The Role of Oxygen Exposure on the Performance of All-Vapor-Processed Perovskite Solar Cells With CuPC Hole Transport Layers. *IEEE J. Photovolt.* **2024**, *14* (5), 758–764. https://doi.org/10.1109/JPHOTOV.2024.3414125.
(41) Harding, A. J.; Dobson, K. D.; Ogunnaike, B. A.; Shafarman, W. N. Thermal and Structural Characterization of Methylammonium- and Formamidinium-Halide Salts. *Phys. Status Solidi A* **2021**, *218* (22), 2100246. https://doi.org/10.1002/pssa.202100246.
(42) Thompson, C. V. Solid-State Dewetting of Thin Films. *Annu. Rev. Mater. Res.* **2012**, *42* (1), 399–434. https://doi.org/10.1146/annurev-matsci-070511-155048.
